# Estimation of the costs of cervical cancer screening, diagnosis and treatment in rural Shanxi Province, China: a micro-costing study

**DOI:** 10.1186/1472-6963-12-123

**Published:** 2012-05-24

**Authors:** Ju-Fang Shi, Jun-Feng Chen, Karen Canfell, Xiang-Xian Feng, Jun-Fei Ma, Yong-Zhen Zhang, Fang-Hui Zhao, Rong Li, Li Ma, Zhi-Fang Li, Jie-Bin Lew, Yan Ning, You-Lin Qiao

**Affiliations:** 1 Department of Cancer Epidemiology, Cancer Institute, Chinese Academy of Medical Sciences, Peking Union Medical College, 17 South Panjiayuan Lane, Beijing, Chaoyang District, 100021, China; 2 Cancer Research Division, Cancer Council NSW, 153 Dowling Street, Woolloomooloo, 2011, NSW, Australia; 3 School of Public Health, University of Sydney, Sydney, NSW, 2006, Australia; 4 Dalian Medical University, 9 Western Section, Lvshun South Street, Dalian, Liaoning, Lvshunkou District, 116044, China; 5 Changzhi Medical College, 161 East Jiefang Street, Changzhi, Shanxi, 046000, China; 6 Xiangyuan Women and Children Hospital, 109 Taihang Road, Xiangyuan, Shanxi, 046200, China; 7 Shanxi Cancer Institute/Hospital, 3 Zhigongxin Street, Taiyuan, Shanxi, 030013, China

## Abstract

**Background:**

Cost estimation is a central feature of health economic analyses. The aim of this study was to use a micro-costing approach and a societal perspective to estimate aggregated costs associated with cervical cancer screening, diagnosis and treatment in rural China.

**Methods:**

We assumed that future screening programs will be organized at a county level (population ~250,000), and related treatments will be performed at county or prefecture hospitals; therefore, this study was conducted in a county and a prefecture hospital in Shanxi during 2008–9. Direct medical costs were estimated by gathering information on quantities and prices of drugs, supplies, equipment and labour. Direct non-medical costs were estimated via structured patient interviews and expert opinion.

**Results:**

Under the base case assumption of a high-volume screening initiative (11,475 women screened annually per county), the aggregated direct medical costs of visual inspection, self-sampled *care*HPV (Qiagen USA) screening, clinician-sampled *care*HPV, colposcopy and biopsy were estimated as US$2.64,$7.49,$7.95,$3.90 and $5.76, respectively. Screening costs were robust to screening volume (<5% variation if 2,000 women screened annually), but costs of colposcopy/biopsy tripled at the lower volume. Direct medical costs of Loop Excision, Cold-Knife Conization and Simple and Radical Hysterectomy varied from $61–544, depending on the procedure and whether conducted at county or prefecture level. Direct non-medical expenditure varied from $0.68–$3.09 for screening/diagnosis and $83–$494 for pre-cancer/cancer treatment.

**Conclusions:**

Diagnostic costs were comparable to screening costs for high-volume screening but were greatly increased in lower-volume situations, which is a key consideration for the scale-up phase of new programs. The study’s findings will facilitate cost-effectiveness evaluation and budget planning for cervical cancer prevention initiatives in China.

## Background

The burden of cervical cancer in China is substantial, with an estimated ~27,000–130,000 cases in 2010, projected to increase to 42,000–187,000 in 2050, in the absence of any preventative intervention [1]. Although there may be considerable heterogeneity within China, the evidence is consistent with a higher burden of disease in some rural areas [1]. Although most women do not have access to screening, a cervical cancer screening initiative has been established for rural women, involving visual inspection with acetic acid (VIA) and cytology. This initiative, which began in 2009, is planned to eventually cover up to “10 million” rural women aged 35–59 years in China [2]. Cost-effectiveness analyses (CEA) of various cervical cancer prevention strategies, including the use of primary screening for the presence of the human papillomavirus (HPV) with a rapid-throughput technology (*care*HPV, Qiagen MD, USA) in rural China have previously been conducted [3-5].

Cost estimation is a central feature of health economic analyses [6] and has a direct impact on the CEA outcomes. In principle, the results of costing studies from different approaches (micro-costing verses hospital charge estimates) and perspectives (societal verses health provider) may vary as a function of the costing study methodology. In many previous health economic studies related to cervical cancer prevention in less developed settings, cost data have been reported as part of broader cost-effectiveness analyses [3,5,7-9]. Some costing studies conducted in other developing countries have reported information on cervical screening and diagnosis; but very little quantification of the costs related to treatment for cervical pre-cancer and cancer has been performed [10-12]. A few local groups in China have estimated cervical cancer treatment expenses, mainly based on charge records from hospitals in urban areas [13-16]. The most precise costing approach, the micro-costing method [6], has not been used extensively in studies in China, either in relation to costing studies for cervical cancer prevention, or more broadly for other diseases.

The objectives of the current study were to use a micro-costing approach, and from a societal perspective, to estimate the aggregate costs of various cervical screening tests that are potentially feasible in low resource settings [5], as well as diagnostic procedures and treatment in rural China. This analysis was conducted as a part of the “National Key Technology R&D Program in the 11th Five-Year Plan” project (“11^th^-5” project), which is a multi-centre epidemiologic and health economic study project supported by the Chinese Ministry of Science and Technology. More than 12,500 women have been screened through this project, using cytology or HPV DNA-based screening for urban areas, and visual inspection or rapid lower-cost HPV DNA testing for rural areas. The “11^th^-5” project was conducted as a research component of a larger visual inspection based screening initiative (Early detection and early treatment of cervical cancer program, EDETCC) [17].

## Methods

### Screening strategies

We considered those cervical screening tests potentially feasible in rural areas in China, including VIA only, VIA combined with visual inspection with Lugol’s iodine (VILI), and self- or clinician-sampling with *care*HPV [18] for primary HPV screening. Visual screening appears to be a simple, safe, acceptable and inexpensive approach but there are substantial issues related to test accuracy and repeatability of visual screening, and screening with visual inspection was not found to decrease cervical cancer mortality in a large-scale trial in rural India [19]. *Care*HPV is a recently developed, rapid-throughput lower cost test for HPV, and has been demonstrated to have high sensitivity for cervical high-grade lesions in a rural Chinese setting; this was 90% for cervical specimens and 81% for vaginal specimens, where vaginal sampling represents the potential accuracy of self-sampling [18]. Women with positive screening results were assumed to be referred for further diagnosis, including colposcopy examination, and biopsy and endocervical curettage (ECC) if indicated. Additional file 1: Table S1 provides more details of the screening and diagnostic pathways which were assumed; these were extensively discussed with local key opinion leaders before the commencement of the costing study [4,5]. Cytology screening was not considered in the current study, which was consistent with the fact that cytology based screening was not recommended by an International Agency for Research on Cancer (IARC) populations with limited resources [20].

### Selection of study sites

Data collection for the current study was conducted in a county hospital and a prefecture hospital in rural Shanxi Province, which is in central north China, from April 2008 to April 2009. Shanxi Province has a total population of 33 million and a geographical area of 156,800 km^2^[21], and the province had a gross domestic product (GDP) per capita of US$2,975 in 2008 [22]. Several important studies and projects related to cervical cancer prevention have been conducted in Shanxi over the last decade, most of the studies being conducted in collaboration with the Cancer Institute of the Chinese Academy of Medical Sciences (CICAMS) [23].

When choosing the sites for the current costing study, the local situation and health systems were considered. There are several administrative levels operating in China, including those at the province, prefecture, county, township and village levels (the smallest unit). County-level hospitals in rural China would potentially be able to provide centralised cervical screening, and some diagnostic and treatment services. For treatment of patients with diagnostically confirmed cervical intraepithelial neoplasia (CIN) or cervical cancer, most of the county hospitals provide only basic surgical facilities, and more comprehensive treatments are available only in hospitals at a higher level (such as prefecture hospitals). Given the particular situation locally, we assumed that a local screening program would be organized to run through a county hospital, and that women would travel to the county hospital for screening with HPV or visual inspection-based screening. For *care*HPV screening, an additional option was considered; it was assumed that a mobile screening team based at the county hospital would visit a township or village and that women would perform self-sampling for HPV testing [5]. For all screening strategies, we assumed that women with confirmed abnormalities travelled either to a county hospital or a prefecture hospital for further treatment. Therefore, screening and diagnosis cost data were collected in the Women and Children’s Hospital (county-level) in Xiangyuan County, which is one of 13 counties of Changzhi Prefecture in Shanxi, with a population of 250,000 with 47,750 women aged 30–59 years in 2000 [21]. For the collection of treatment cost data, we extended the survey to an affiliated hospital of the Changzhi Medical College (prefecture-level, located in Changzhi City, the capital city of Changzhi Prefecture, which has a population of 3.16 million [21]).

### Overview of costing methodology

**Figure 1:**
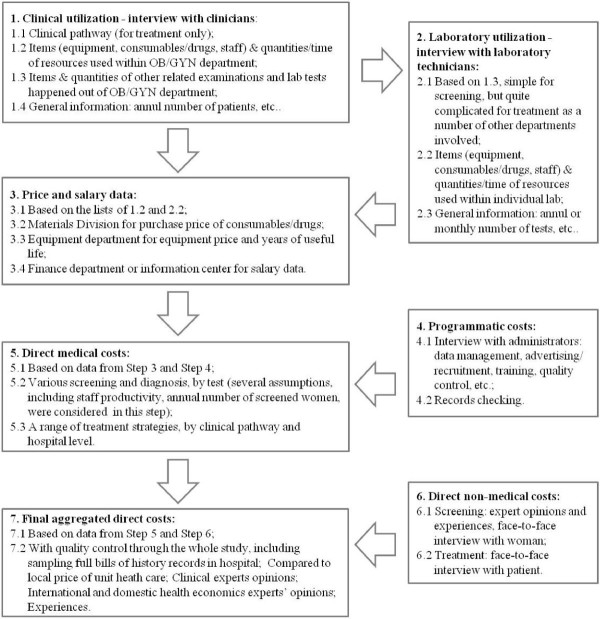
**Workflow of costing study in Shanxi field†.** † Study sites: The Women and Children Hospital of Xiangyuan County, and affiliated hospitals of Changzhi Medical College in Shanxi Province, 2008–2009.

Direct medical and non-medical costs related to cervical screening, diagnosis and treatment of cervical high-grade lesions and cervical cancer were collected in this study. Figure 1 shows details of the cost collection process. Direct medical costs associated with clinic visits and laboratory tests were identified using observational field work and extensive consultation with experienced local medical staff. The field workflow commenced with interviews with local clinicians, followed by interview with lab technicians, from which lists of items and the quantities consumed were generated. Costs of all individual items and salary data were then collated, along with the collected quantity data, to generate the final aggregated unit costs. For each individual clinical visit and laboratory test, the following items were included in the costing exercise: consumables (quantity used and unit price), drugs (quantity used and unit price), equipment (quantity used, price, years of useful life, number of cases processed annually), and staff (staff category, working time breakdown). Data sources for direct non-medical cost estimates included structured patient interviews and expert opinion. Each item was costed at 2008 prices, which were collated from financial records at each hospital. At least 15 experts or experienced field staff were interviewed at each hospital; these included clinicians, laboratory technicians, and administrative staff. All cost data were originally calculated in Chinese Yuan (CNY) and presented in US dollars (exchange rate: 1 US$ = 6.8304 CNY; 19 May 2009). For equipment depreciation, we used a discount rate of 3% to derive annual costs [3,10].

When estimating equipment costs, we assumed that the years of useful life was 7–10 years. We used enhanced annual volume and a discount (depreciation) rate of 3% to obtain the average equipment cost per examination per woman. Usually, the costs relating to use of equipment for colposcopy, biopsy, ECC and other diagnostic procedures were considerably more expensive than the local labour cost. For example, the cost of a colposcope used in the current study was ~ $17,600, but the annual net income per resident in rural China was only $697 in 2008 [24]. Based on these assumptions, if a larger number of women are screened and referred for diagnosis, the average equipment cost allocation for each woman would become lower, while the remaining components (labour and supplies) of the aggregated costs would remain unaltered.

### Direct medical costs of cervical screening and diagnosis

In addition to considering of the costs associated with clinical visits and laboratory processes, we also considered programmatic costs including data management, advertisements on local radio, dissemination of leaflets, staff training and quality control, and staff transportation. These costs were allocated on a per-woman basis. For visual screening tests, we assumed that the ongoing quality control and training cost was 23% of other clinical costs, based on the average percentage used in a previous study in five developing countries [7]. A lower percentage for the ongoing quality control/ training cost (10%) was assumed for *care*HPV, colposcopy, biopsy and, ECC procedures (including histology) because of their assumed higher stability and reproducibility when compared to visual inspection. We assumed all screening programs would be implemented at established hospital sites and therefore did not take into account the cost of real estate or hospital buildings, power, water or other aspects of the general infrastructure.

Based on previous field experience from prior studies, we assumed that a maximum of 60 women could be screened on average each day by a well-trained screening team operating at full workload. This corresponds to a total of 11,475 women screened annually (at 75% of the full workload, over 255 working days). However, in order to account for lower volume screening situations or for operational scale-up of large programs, we also examined the impact of alternate lower volume screening assumptions, and assumed that (i) 2,000 and (ii) 6,000 women were screened annually in a sensitivity analysis. These alternative lower volume assumptions were based on previous experience involving a single screening team working on a part time basis since 2004 to implement a screening demonstration project and the EDETCC of visual inspection based screening which was conducted in Xiangyuan County, Shanxi Province.

In clinical field studies in Shanxi Province, the screening test positivity rate has been reported as 10.7% for visual inspection and 14.6% (in women aged 30–54 years) [25] or 14.5% (in women aged 15–59 years) [26] for HPV testing. Based on these data, we used 14.6% test positivity as broadly representative of field performance in Shanxi for all tests. All women with positive primary screening results were assumed to be referred to further colposcopy examination [25]. Based on field studies we also assumed that 55% [27] and 20% [28] of women undergoing colposcopy would then undergo cervical biopsy and ECC, respectively. Thus, the annual numbers assumed in the base case, for women who received colposcopy, biopsy and ECC in the current analysis were 1,675, 925 and 335, respectively. Staff salary data were obtained from the two study hospitals. The unit cost for the *care*HPV test in the base case was assumed to be US$5 (which is the targeted price by Qiagen for mass screening programs supported by the public sector in low-resource countries [3]), and which was assumed to include test-specific consumables-such as cervical sampler, storage medium and equipment costs. Other cost items related to *care*HPV processing, including generic consumables and staff costs, were evaluated separately.

### Direct medical costs of treatment of cervical precancer/cancer

We also obtained costs related to treatment for cervical precancerous lesions or invasive cervical cancer. The micro-costing approach was used jointly with a clinical pathway approach in order to reduce missing items. It should be noted that the clinical pathways in this context are all from the local setting. As Figure 1 shows, the gynaecological staff members were interviewed to identify the bed days (if any), procedures and events involved in each treatment modality. They were also interviewed to identify the consumption of clinical supplies/drugs, equipment, staff and time, laboratory tests, and other examinations that occurred in each individual day for each treatment type. Interviews were then extended to medical professionals in other departments of the hospital, including the surgery and anaesthesia units, radiotherapy/chemotherapy, pathology and other examinations (such as electrocardiogram, ultrasonic and chest x-ray facilities). Additional file 1: Table S1 gives further details on the clinical pathways for individual treatment types, including bed days, number of examinations/tests involved, type of anaesthesia, and local post-treatment follow-up strategies.

### Direct non-medical costs of cervical screening and diagnosis

For screening and diagnosis, the direct non-medical costs included women’s average time in seeking and receiving care and two-way transportation expenses. For screening conducted at the county hospital, women were assumed to take a public bus to the hospital in which the primary screening was conducted. We calculated the average area of 119 counties in Shanxi Province (1,316 km^2^), and then used half the radius of the (assumed circular) county area ($\text{Length}=(\sqrt{\text{Country Area}/\pi )}/2$) to estimate the average distance between a woman’s home and the county hospital, which was calculated to be 10 km. For the mobile screening program (involving testing with self-sampling *care*HPV only), women were assumed to collect the vaginal specimens in a local clinic at their home township or village ( Additional file 1: Table S1); in such a situation, we used half the radius of the average township area of Xiangyuan County (105 km^2^) to estimate the average distance between place of residence and the township/village clinic, which was calculated to be 3 km. Taking into account the average speed of a local public bus of 20 km per hour, this information was used to estimate a woman’s two-way transportation time. A woman’s waiting time for a public bus was estimated based on expert opinion. Women’s waiting time in the hospital/clinic was estimated by experienced staff in the field. Information on women’s time in examination and their out-of-pocket expense for two-way transportation was obtained from experienced investigators involved in previous local projects and informed by inspection of the ongoing screening process. The annual net income per capita for residents in rural China ($697 in 2008) [24] was applied to convert women’s time into equivalent earnings loss, assuming 255 working days and 8 working hours per day. The final average calculated costs per woman in earnings loss were then $2.73 per day or $0.00586 per minute.

### Direct non-medical costs of treatment of cervical precancer/cancer

For estimating the direct non-medical costs related to cervical precancer or cancer treatment, we used categories for costs based on the recommendation by World Health Organization, such as women’s time and transportation [29]; but we also included further categories (such as patients and their carer’s additional out-of-pocket expenses on transportation, accommodation and food) and other out-of-pocket expenses (such as women and their carer’s time in seeking/receiving care, and post-treatment recovery time) [6]. A face-to-face interview was conducted with local trained doctors and nurses, and among a convenience sample of women who attended the 11^th^-5 project, and among women who were receiving treatment in county or prefecture hospitals. A total of 108 patients with cervical pre-cancer or cancer were interviewed. Thirty of these women were from county hospitals (12 with CIN2, 17 with CIN3, and 1 with invasive cervical cancer), and 78 were from prefecture hospitals (25 with CIN3, 3 with micro-invasive cancer, and 50 with invasive cervical cancer).

### Quality control of the costing exercise

We undertook a range of activities to reduce the chance of missing any items in the costing exercise. Firstly, interviews of local clinicians and technicians were restricted only to experienced and senior staff. Secondly, in order to check the information obtained from the interviews, at least one hospital record for each possible treatment type from the local hospitals was checked by CICAMS researchers to confirm inclusion of all relevant items. Thirdly, the preliminary aggregated data were compared to the local prices for various heath care procedures when they were available [30], and outlier results were given additional attention. In addition, the close-to-final aggregate results were sent to local health economists and clinicians for comments. There were multiple interactions between the CICAMS team and the field staff.

### Main outcomes

The aggregated unit costs for each screening and diagnostic test, and for each treatment type (according to the level at which treatment was performed – county or prefecture) were calculated. Component costs were calculated in two ways: firstly, broken down by the costs of clinical visit/hospital care, laboratory and programmatic costs; and secondly broken down by costs of drug/supplies, equipment and staff. Women’s out-of-pocket expenses, the related time spent on seeking/receiving care and post-treatment recovery, and the consequent aggregated direct non-medical costs of screening, diagnosis and treatment were also estimated.

In sensitivity analysis, we particularly focused on the role of screening volume, because this is a parameter of major uncertainty and, a priori, was likely to have a major impact on aggregate costs due to the potential for economies of scale at high volumes. We also included a number of other key parameters (discount rate, the annual number of screened women, screening positivity rate, biopsy/ECC rate, and programmatic costs) in one-way sensitivity analysis.

### Ethical approval

The costing study was conducted as one component of a larger government-sponsored cervical screening project (EDETCC), which was approved by the Institutional Review Board of the Cancer Foundation of China.

## Results

### Direct medical costs of cervical screening and diagnosis

**Figure 2:**
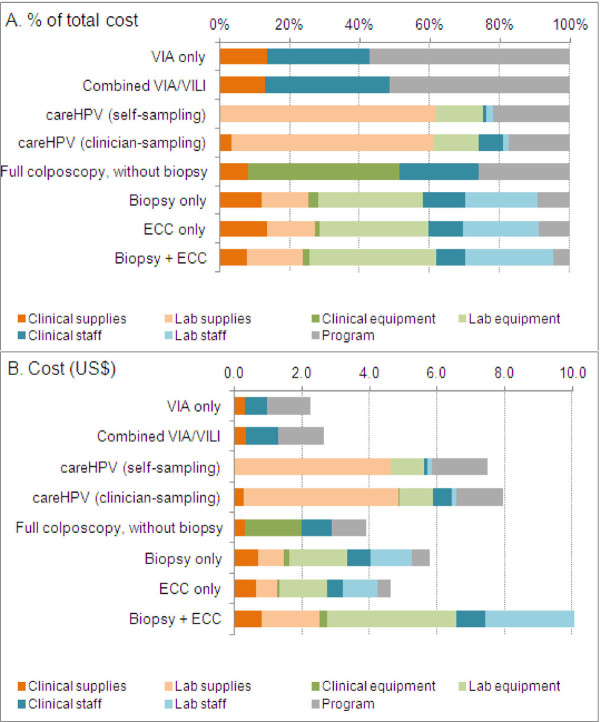
The proportional breakdown of costs of cervical screening and diagnosis.

Under the base case assumption that screening was conducted at full volume (11,000 women screening per annum in a county), the aggregated direct medical costs of screening were found to be $2.24, $2.64, $7.49 and $7.95 for VIA only, combined VIA/VILI, *care*HPV (self-sampling) and *care*HPV (clinician-sampling), respectively. The direct medical costs of diagnostic procedures were found to be $3.90, $5.76, $4.63 and $10.61 for full colposcopy (with no biopsy), biopsy only, ECC only, and biopsy combined with ECC, respectively. Unsurprisingly, the laboratory costs (including test kit cost) were found to comprise a major component (>60%) of the total aggregated cost of *care*HPV screening and of the pathology tests (biopsy, ECC) (Table 1). It was also found that the programmatic costs (including data management, advertisements, recruitment and staff training) ranked as the largest component of the aggregated cost of visual inspection screening (Table 1). This is explained by relatively low labour, equipment and consumable costs, leading to a low clinical visit cost for this screening test. More information on the component costs, broken down by clinical visit, laboratory and programmatic costs are presented in Table 1. Figure 2 shows a detailed analysis of the costs broken down according to supplies, equipment and staff time. It was found that clinical and laboratory staff labour costs did not comprise a major proportion of the total cost for most procedures: the exception was visual inspection screening for which 29–36% of the aggregated cost was directly related to labour.

**Table 1:** Direct medical costs of cervical screening and diagnosis (US$)

	**Clinical visit cost**	**Laboratory cost**	**Programmatic cost†**	**Aggregated cost**
VIA only	0.96	0.00	1.28	2.24
Combined VIA/VILI	1.28	0.00	1.36	2.64
*care*HPV (self-sampling)	0.10	5.75	1.64	7.49
*care*HPV (clinician-sampling)	0.81	5.75	1.39	7.95
Full colposcopy, without biopsy	2.88	0.00	1.02	3.90
Biopsy only	1.55	3.68	0.53	5.76
ECC only	1.14	3.07	0.42	4.63
Biopsy + ECC	1.90	8.18	0.53	10.61

† The programmatic cost included staff transportation (for *care*HPV self-sampling strategy only), advertising/ recruitment, training/ quality control, and data management.

**Figure 3:**
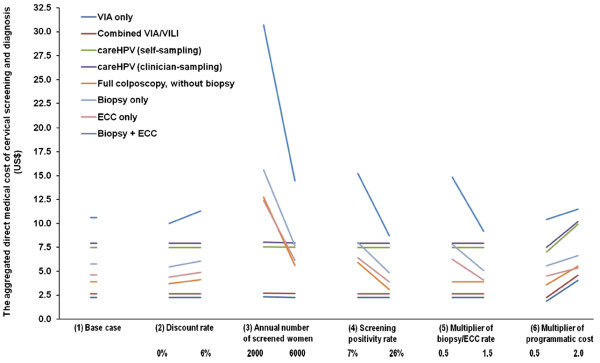
**One-way sensitivity analysis (SA) for aggregated costs of screening and diagnosis†.** † (1) Base case assumptions included: a discount rate of 3% [3,10], 11,475 women screened annually, a screening positivity rate of 14.6%, a cervical biopsy rate of 55% [27] and a ECC rate of 20% [28] for women with colposcopy. Various programmatic costs (including costs of advertising/ recruitment, training/quality control, and data management) ranged from $0.42 to $1.64. (2) SA for discount rate: 0%–6%; (3) SA for annual number of screened women: 2,000 and 6,000, based on previous experience, as explained in Methods. (4) SA for screening positivity rate: 7% [31] and 26% [32], two screening positivity rates observed in populations in China. (5) SA for multiplier of biopsy/ECC rate: because biopsy rate is usually clinician-dependent and subject to the underlying prevalence of disease in the population, we multiplied the rates at baseline for SA, as a function of multiplier of the biopsy/ECC rate at baseline (0.5, 1.5). (6) SA for multiplier of programmatic cost: the programmatic costs we used for baseline analysis were halved and doubled (0.5, 2.0), to account for the relatively large uncertainty associated with this parameter.

Figure 3 presents the findings of the sensitivity analysis for the aggregated costs of screening and diagnostic procedures. The aggregate costs for diagnostic procedures were found to be sensitive to assumptions related to the annual number of screened women. When only 6000 women were assumed to be screened annually, the aggregate costs of colposcopy, biopsy only, ECC only, and ECC combined with biopsy, increased to $2.26 $5.61, $7.66, $6.13 and $14.48 (~1.4 times the base case costs), respectively. These aggregate costs further increased to $12.76, $15.61, $12.41 and $30.70 (~3 times the base case costs), respectively, when the number of screened women was assumed to be to 2,000 per annum. Taking colposcopy as an example to estimate the contribution of equipment costs to the aggregated diagnostic costs, when 11,475, 6,000 or 2,000 women were screened annually, the equipment cost per examination per woman was found to be $1.68, $3.22 or $9.66, which comprised 43.2%, 57.4% or 75.7% of the aggregated costs of the colposcopy examination, respectively. By contrast, the aggregate costs of screening procedures were relatively robust to screening volume (<1% variation when assuming 6,000 screened women per annum, and <5% variation assuming 2,000 screened women per annum). The results for the aggregate costs of both diagnostic and screening procedures were relatively robust to changes in the assumed discount rate, screening positivity rate, biopsy/ECC rate, and programmatic costs (Figure 3).

### Direct medical costs of treatment of cervical pre-cancer/cancer

**Figure 4:**
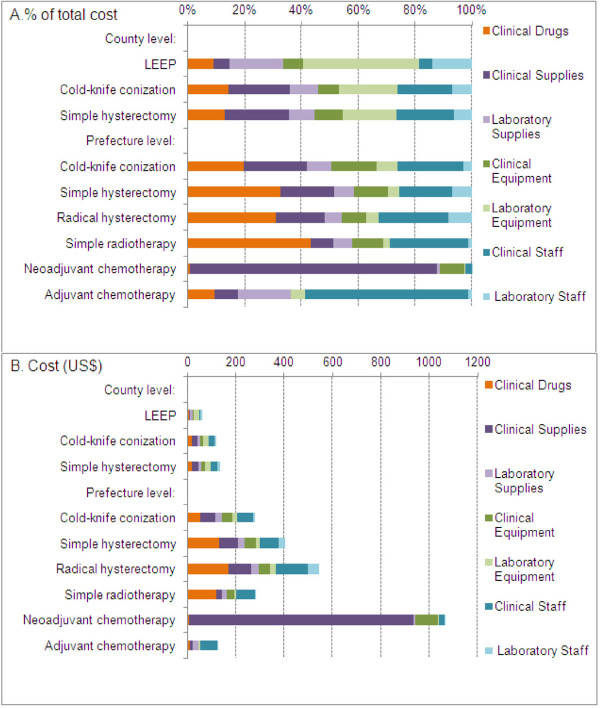
The proportional breakdown of costs of treatment cervical precancer/cancer.

In the county hospital setting, the total aggregated direct medical costs for the three available treatments for cervical pre-cancer/cancer were found to be $61.38, $120.85 and $133.42 for loop electrosurgical excision procedure (LEEP), cold-knife conization (CKC), and simple hysterectomy, respectively. In the prefecture hospital setting, LEEP is not generally performed; and the total direct medical costs were estimated as $280.75, $404.73 and $544.39 for CKC, simple and radical hysterectomy, respectively, and were $281.65, $1,064.05, and $125.46 for radiotherapy, neoadjuvant chemotherapy and adjuvant chemotherapy, respectively. The proportional costs related to laboratory expenses in the prefecture hospital setting (<25% for all procedures) were lower than that in the county hospital (>34%). Table 2 shows further information for the components of the treatment costs, broken down into categories of clinical costs and laboratory costs related to cervical precancer or cancer treatment. The proportional breakdown of drugs, supplies, equipment and staff suggests that, compared to the prefecture hospital, the county hospital spent proportionally less on drugs (<14% vs. >20%), but slightly more on equipment (>28% vs. <23%) (Figure 4a). The costs obtained from the prefecture hospital were generally found to be higher than those incurred in the county hospital, for comparable treatment types, and the differences between were mainly derived from more extensive use of clinical supplies, increased labour costs and drugs in the prefecture hospital (Figure 4b).

**Table 2:** Direct medical costs of treatment cervical precancer/cancer (US$)†

		**Clinical cost**	**Laboratory cost**	**Aggregated cost**
A.County level					
	Loop electrosurgical excision procedure (LEEP)	16.08	45.30	61.38	
	Cold-knife conization	75.55	45.30	120.85	
	Simple hysterectomy	88.12	45.30	133.42	
B. Prefecture level					
	Cold-knife conization	228.01	52.74	280.75	
	Simple hysterectomy	332.16	72.57	404.73	
	Radical hysterectomy	444.35	100.03	544.39	
	Simple radiotherapy	251.90	29.75	281.65	
	Neoadjuvant chemotherapy	1,051.74	12.31††	1,064.05	
	Adjuvant chemotherapy	93.89	31.56††	125.46	

† LEEP treatment was not available in the surveyed prefecture hospital.

†† Related to bloods tests and ultrasound examinations.

### Direct non-medical costs of cervical screening and diagnosis

Table 3 shows the aggregate direct non-medical costs for screening and diagnostic procedures. The final aggregate direct non-medical cost ranged from $0.68 (for self-sampled *care*HPV screening) to $2.27 (for full colposcopy with biopsy and/or ECC). Generally, it was found that for most procedures, women spent less time interacting with clinicians than they did on transportation and waiting times. The self-sampled *care*HPV screening procedure was associated with the lowest direct non-medical cost due to savings in transportation time and out-of pocket expenses.

**Table 3:** Direct non-medical costs of cervical screening and diagnosis†

	**Women’s average time in seeking and receiving care (minutes)**			**Two-way transportation (US$)**	**Total (US$)**
	**Two-way transportation**	**Waiting**	**Receiving care**		
VIA screening	120	40	15	1.17	2.16
Combined VIA/VILI screening	120	40	21	1.17	2.20
*care*HPV screening (self-sampling)	40	20	8	0.29	0.68
*care*HPV screening (clinician-sampling)	120	40	13	1.17	2.15
Full colposcopy, without biopsy or ECC	120	40	19	1.17	2.19
Full colposcopy, with biopsy or ECC	120	40	34	1.17	2.27

† Data sources: Local screening demonstration projects, expert opinion, the annual net income per capita in rural China ($697 in 2008) [24]; Women with positive self-sampling *care*HPV results were referred for a colposcopy/biopsy in county hospital another day. Most of the items related to time spent and two-way transportation out-of-pocket expenses were the same when an immediate colposcopy followed the primary screening test (except for a few items). For primary screening tests followed by colposcopy with or without biopsy/ECC on the same day, an extra 7 minutes or 22 minutes were needed for women going through the diagnostic procedure(s). For those received clinician-sampling *care*HPV primary screening, an extra 150 minutes were needed for waiting for a notification of whether further colposcopy examination on the same day was required.

### Direct non-medical costs of treatment of cervical pre-cancer/cancer

The aggregate direct non-medical costs associated with treatment ranged from $83 to $494 (Table 4). A wide variation was observed in the reported costs, which may be in part due to the difficulties experienced in conducting face-to-face interviews with less educated women these difficulties may have increased the potential for recall bias.

**Table 4:** Direct non-medical costs of treatment cervical precancer/cancer

		**Number of study subjects**	**Out-of-pocket expenses (US$)**		**Related time spent† (Days)**		**Total cost (US$)**	
			**Median**	**Interquartile range**	**Median**	**Interquartile range**	**Median**	**Interquartile range**
A. County level								
	Cold-knife conization	14	34	15–38	18	7–46	83	34–164
	Simple hysterectomy	11	147	67–293	127	40–210	494	177–867
	Radical hysterectomy	5	53	34–94	60	28–276	217	110–807
B. Prefecture level								
	Cold-knife conization	1	98	-	25	-	166	-
	Simple hysterectomy	19	146	79–234	53	32–109	291	167–532
	Radical hysterectomy	51	109	78–199	67	38–124	292	181–538
	Simple radiotherapy	7	110	75–706	52	42–161	252	189–1146

† Including self-reported women and their carer’s time in seeking/receiving care, and post-treatment recovery time at home; cost per woman in earnings loss was $2.73 per day.

## Discussion

The present analysis provides a range of calculated aggregated costs for cervical screening, diagnostic procedures and the treatment of cervical precancerous lesions and invasive cervical cancer in rural China. We found that the scale on which future cervical screening initiatives will be conducted (i.e. the volume of women screened) will have a major impact on the diagnostic costs in this setting, and we have quantified this effect in detail. The diagnostic costs are comparable to screening costs in situations involving high-volume screening, but greatly increased in lower-volume settings, which is a key consideration for the scale-up phase of new screening programs. The findings of this study can be applied to inform future budget planning and cost-effectiveness evaluations of planned potential large-scale cervical screening programs in rural China.

When considering the *care*HPV test, we found that the self-sampling method is slightly less expensive than the physician-sampling method, as expected, due to savings in clinician time. However, the overall savings due to implementing self- rather than clinician-sampling were relatively modest, of the order of $0.50 or less; this modest saving is because the unit cost of HPV testing (i.e. the cost at which the test is supplied by the manufacturer, assumed to be $5 in this analysis) has more significant impact on the aggregated costs than the associated labour cost does in this setting. As expected, we found that the costs of visual inspection screening tests are lower than those related to *care*HPV screening, because a large proportion (up to 40%) of the aggregated test cost is directly related to labour. However, this advantage in affordability terms should be interpreted in context of the most recent data on the relative efficacy of these screening tests. For example, in a recent study in rural China it was found that the accuracy of HPV test for the detection of CIN2+ is substantially better than for VIA [18]; furthermore, a recent randomised trial in rural India found that there was no significant reduction in the rate of cervical cancer death over eight years after a single round of VIA screening compared to no intervention, whereas a single round of HPV screening was associated with a mortality reduction of approximately 50% [19]. Cost-effectiveness analyses, therefore, are needed to fully represent the full picture of the relative benefits and costs of visual inspection based screening compared to HPV-based screening [5].

The aggregated costs of visual inspection screening and HPV screening from other less developed countries (including India, Kenya, Peru, South Africa and Thailand) have previously been estimated using various costing methods such as quantity-and-price approaches. It is difficult to directly compare the findings of these prior studies with our results, since these costs were estimated in different countries and currencies and for the year 2000 or 2002 [7,9,11]. However, our estimation of the aggregate cost of VIA is substantially lower than that from a recent study conducted in Ghana, in which resource utilisation data were collected from four screening centres; in that study the costs of VIA were found to vary from US$5-15 (at 2009 prices) [12]. In prior studies, labour costs have tended to comprise a major part of VIA costs; for example, personnel costs comprised more than half of the aggregate costs in India, Thailand, Kenya and Ghana [7,9,11,12], whereas in our base case analysis in China we found that this proportion was approximately one third of the total cost. For *care*HPV screening in this rural Chinese setting we found that direct costs related to supplies, equipment, and laboratory processes had a more substantial impact on the aggregated costs than other screening costs, which is consistent with previous work in other developing countries, although it should be borne in mind that in some prior studies the specific use of the *care*HPV technology was not assumed [7,10,11].

The importance of screening scale has also been addressed by the recent Ghanaian study which found that the number of women screened per provider was one of the most important determinants of costs [12]. Our baseline estimate of aggregate test costs was based on high screening volumes, and in this situation we found that the total cost of colposcopy is lower than that of *care*HPV screening. Colposcopy may have relatively low sensitivity compared to HPV DNA screening, but it has been found that colposcopy was more sensitive than visual inspection screening in rural China [27,33]. This raises the possibility that, in a high volume screening situation in this setting, colposcopy may be a viable alternative to HPV testing as a screening test; this has been suggested in the context of other settings [20]. However, this would require further extensive clinical and cost-effectiveness evaluation in this setting before implementation. Our baseline results were derived assuming the annual number of screened women in a program organised at the county level was over 11,000. As an example, if this number of women were screened in Xiangyuan County in rural Shanxi Province, this would represent an estimated population coverage in women aged 30–59 years of approximately 24%. Therefore, under the base case assumption of a high-volume screening initiative, one screening team could implement close to population-wide coverage in the target age group if screening were conducted over a 4–5 year period. Although, it is quite difficult to reach high coverage within organised programs unless effective individual invitation policies are developed, potentially 60% participation on a per-woman basis has been shown to be achievable in this setting [5]. By contrast, if only 2000 women were screened (corresponding to an approximate annual population coverage level of 4% in the target age group), the aggregate cost of the colposcopy examination would be tripled, and would exceed the total cost of *care*HPV. Similarly, the relative cost of histopathology would increase considerably in lower volume screening situations. This is because in this rural setting, the cost of labour is relatively low compared to capital equipment costs. These findings imply that consideration of the scale and scale-up timing of new cervical screening programs in rural China is very important, and may influence considerations of the most appropriate screening test to be implemented.

The current study also estimated the costs related to cervical precancer and cancer treatment in this rural Chinese setting. We found that the direct medical costs of LEEP, CKC and simple and radical hysterectomy varied from $61–$544, depending on the procedure and whether conducted at county or prefecture level, and that direct non-medical expenditure varied from $83 to $494 for pre-cancer/cancer treatment. These findings may be comparable to those presented in a prior estimate which took a broad perspective across 25 countries (including China) estimated the direct medical cost of cervical cancer treatment for China on average as ~1,500 international dollars (I$) in 2005 for cancer stage-I and I$1,700 for stage II–IV cancer, by leveraging available data in select other countries and extrapolating to China based on gross domestic product per capita and some other indicators [8]. The costs obtained from the prefecture hospital ($281–$544) were generally found to be higher than those incurred in the county hospital ($61–$133). Given the relatively low annual net income for rural residents in China (~$700 per capita in 2008) [24], these findings suggest that the treatment of cervical pre-cancer and cancer, particularly treatment in prefecture hospitals, leads to a considerable economic burden for local patients. This raises the possibility that the overall treatment cost could potentially be reduced by treating more patients at county rather than prefecture hospitals; although it is not clear if the cost differentials in treatment would be maintained in a situation of high volume screening with a consequently enlarged referral population for treatment. To develop and strengthen the health care capacity of county hospitals and to enhance their quality control processes would be an important objective for the local health care system.

The micro-costing approach we used in this analysis built on an earlier assessment conducted in 2005 [3], by adding more elements to cost the complete pathway from screening and diagnosis to treatment, and by implementing a full quality control process. We found that CKC is more expensive than LEEP ($120 for CKC, $61 for LEEP), but the earlier study reported very similar values for the two procedures ($87.13 for CKC, $87.58 for LEEP) [3]. Overall, treatment costs from the current study were generally lower than those of the earlier study [3]. Table 5 provides summary information for both studies. Limited data are available on cervical cancer treatment cost estimates based on hospital charge records [14-16] (Table 5). Our micro-costing estimates were found to be relatively lower than those reported by local groups [14-16]. For example, two previous analyses conducted in provincial hospitals reported an average treatment expense per patient with cervical cancer as ~ $1,390 (1997–2001, N = 106) [15], and as ~ $1,464 (2006) and ~ $1,185 (1996–2006, N = 1,564) [16]; and a county-hospital-based cost-benefit study reported values based on expert opinion of ~ $585 for CKC and $1,756 for radical hysterectomy [14]. Our previous work using audits of hospital charges to estimate direct medical costs also found that the average fee per cervical cancer patient was ~ $575 at county level and ~ $1,550 at prefecture level [4,5] (Table 5).

**Table 5:** Comparison of reported cost estimates in relation to cervical cancer for China

**Study**	**Year of estimation (article language)**	**Study population**	**Rural or urban setting**	**Overall study design**	**Cost data collection**	**Perspective**	**Cost category covered**	**Reported cost of select or available items (US$)**
The current study	2008–2009 (English)	Shanxi, Prov.	Rural	Purely costing study	Micro-costing approach (county and prefecture level)	Societal (direct medical and non-medical costs)	Screening, diagnosis and treatment	Indicated in Tables 1, 2, 3, and 4 of the manuscript
Shi et al 2011, Canfell et al 2011 [4,5]	2008 (English)	Shanxi, Prov.	Rural	Cost-effectiveness analysis	Micro-costing for screening, diagnosis and part of treatment (LEEP and simple hysterectomy); Checking audits of hospital charges to estimate cancer treatment costs	Societal (direct medical and non-medical costs)	Screening, diagnosis and treatment	VIA screening: $3.55 (mobile), $4.30 (program-based); VILI screening: $0.40; *care*HPV: $9.20 (self-sampling), $10.34 (provider-sampling); LEEP: $55.95; Cancer treatment: $628 (FIGO I); $1953 (FIGO II); $1810 (FIGO III); $663 (FIGO IV)
Levin et al 2009 [3]	2005 (English)	Shanxi, Prov. (Rural)	Rural	Cost-effectiveness analysis	Micro-costing (county and national level)	Societal (direct medical and non-medical costs)	Screening, diagnosis and treatment	Direct medical costs at a county level: Colposcopy: ~$8.21; Biopsy: ~$15.46; LEEP: ~$87.58; CKC: ~$87.13; Simple hysterectomy: ~$354; Local cancer: ~$387; Regional/distant cancer: ~$1,739
Goldie et al 2005 [8]	2005 (English)	25 Asia Pacific regions, including China	Not indicated	Broad cost- effectiveness analysis	Leveraged available data in select other countries and extrapolate to China based on several indicators, including gross domestic product per capita	Societal	Screening, diagnosis and treatment	Average direct medical cost of cervical cancer treatment: ~I$1,500 for cancer stage I, ~I$1,700 for cancer stage II–IV
Deng et al 2010 [14]	2006 (Chinese)	A county-level site (Liuyang) in Hunan Prov.	Rural	Cost-benefit analysis	Standard hospital charges (released by the local Office of Health of Hunan Prov.) for screening and diagnosis; expert opinion for treatment	Medical cost only	Screening, diagnosis and treatment	Pap smear screening: ~$4; Colposcopy: ~$13; Biopsy (one punch): ~$18; CKC: ~$585; Radical hysterectomy: $1,756; Mainly chemo/ radiotherapy: ~$3,658
Wang et al 2009 [16]	1996–2006 (Chinese)	Lanzhou City, Gansu Prov.	Urban	Time-trend analysis of in-patient hospitalization fee (cervical cancer and breast cancer)	Hospital bill audits review (N = 1,564)	In-patient fee only	Cancer treatment only	Average treatment expense per patient with cervical cancer: ~$1,464 (2006) and ~ $1,185 (1996–2006)
Guan et al 2004 [15]	1997–2001 (Chinese)	Wuhan City, Hubei Prov.	Urban	Time-trend analysis of in-patient hospitalization fee (cervical cancer only)	Hospital bill audits review (N = 106)	In-patient fee only	Cancer treatment only	Average treatment expense per patient with cervical cancer: ~$1,390 (1997–2001)
Tan et al 2003 [13]	1996–2001 (Chinese)	Beijing City	Urban	Analysis of in-patient hospitalization fee and clinical outcomes (Hysterectomy only)	Hospital bill audits review (N = 4,180)	In-patient fee only	Treatment only	Average treatment expense of various hysterectomy types: ~$736 - ~ $1,094

This analysis has several limitations. Firstly, the study was conducted in a county-level and a prefecture–level hospital, but in actuality, some cervical cancer patients from rural areas travel to provincial hospitals for treatment, and we were not able to quantify costs in this setting. Secondly, we did not take into account the costs of minor or major related complications, and this issue potentially has greater impact on the treatment cost of cancer patients in more advanced cancer stages. Thirdly, as previously described, we have not included all the overhead costs and post-treatment follow-up costs in the final aggregate cost calculation. Fourthly, the treatment costs of the current study are presented by treatment type, rather than by cancer stage (used by most cervical cancer prevention cost-effectiveness studies), although these can be converted to costs by stage if local data on treatment patter-of-care by stage are available. Fifthly, it is not possible in a direct costing study such as this to directly assess the impact of a new screening program on the future quality of cancer treatment. Sixthly, the absence of costs related to cytology is also one of the limitations of the current study. Furthermore, it should be noted that the screening and diagnosis costs presented in the current study, should be regarded as conservative (low-end) estimates, considering the high-volume screening we assumed in the baseline estimate.

Two recently published cost-effectiveness evaluations have utilised preliminary data for screening and diagnostic costs which were obtained from the present costing study. The first of these evaluated the cost-effectiveness of different screening technologies and approaches in rural Shanxi Province [5]. Assuming high screening volumes, it was found that the cost-effectiveness of primary *care*HPV screening compares favourably to that for visual inspection screening methodologies [5]. The data have also been used in analysis of the cost-effectiveness of combined screening and HPV vaccination approaches in rural China [4]. In the absence of detailed data on HPV vaccine costs in this setting, this analysis took a threshold approach to identify the cost per vaccinated girl (CVG) at which HPV vaccination would become a cost-effective alternative or addition to screening women with *care*HPV. We found that strategies involving vaccination would be cost-effective at CVGs of US$50–54 or less, but at CVGs > $54, screening-only strategies would be more cost-effective [4].

## Conclusions

To our knowledge, the current study is the most detailed analysis of cervical cancer screening, diagnostic and treatment procedure costs in rural China. We used a micro-costing approach and took a societal perspective in order to perform a comprehensive evaluation. These results suggest that diagnostic costs were comparable to screening costs for high-volume screening but were greatly increased in lower-volume situations, which is a key consideration for the scale-up phase of new programs. Our estimates will be applicable to further cost-effectiveness evaluations of new cervical cancer prevention strategies, and will be critical for budget planning, for local government policy makers in China.

## Abbreviations

CEA, Cost-effectiveness analysis; CICAMS, Cancer Institute of Chinese Academy of Medical Sciences; CIN, Cervical intraepithelial neoplasia; CKC, Cold-Knife Conization; CNY, Chinese Yuan; ECC, Endocervical curettage; FIGO, International Federation of Gynecology and Obstetrics; FU, Follow-up; GDP, Gross domestic product; HPV, Human papillomavirus; LEEP, Loop electrosurgical excision procedure; SA, Sensitivity analysis; VIA, Visual inspection with acetic acid; VILI, Visual inspection with Lugol’s iodine.

## Competing interests

The authors declare that they have no competing interests.

## Authors’ contributions

JFS, JFC, KC and YLQ were responsible for the study concept and design. JFS, RL, JFC, XXF, JFM, YZZ, FHZ, LM, ZFL and YN collected the data from field studies. JFS, KC, JFC, XXF and YLQ analysed and interpreted the data. JFS and KC drafted the manuscript. JFC, XXF, JBL and YLQ revised the manuscript. All authors read and approved the final manuscript.

## Pre-publication history

The pre-publication history for this paper can be accessed here:

http://www.biomedcentral.com/1472-6963/12/123/prepub

## Supplementary Material

Additional file 1: Table S1. Description of cervical screening, diagnosis and treatment. Click here for file
