# Supplementary material for: Estimation of the costs of cervical cancer screening, diagnosis and treatment in rural Shanxi Province, China: a micro-costing study
Source: BMC Health Serv Res. 2012 May 24;12:123. doi: 10.1186/1472-6963-12-123 (PMC3461448; doi:10.1186/1472-6963-12-123)
Supplement: Additional file 1: Table S1. — Description of cervical screening, diagnosis and treatment. [file 1472-6963-12-123-S1.doc]

**Appendix Table 1 Description of cervical screening, diagnosis and treatment**

| **A. Screening & diagnosis at county level** | | **Special major item needed?** | **Sample laboratory procedure**  **needed?** | **Management procedure after positive result** | | **Additional details** | |
| --- | --- | --- | --- | --- | --- | --- | --- |
|  | VIA only | No | No | Immediate colposcopy | |  | |
|  | Combined VIA/VILI | No | No | Immediate colposcopy | |  | |
|  | *care*HPV (self-sampling) | Yes, supplies and equipment | Yes | Colposcopy on next visit | | Screening teams from the county hospital travelled to villages/township to guide the local woman for vaginal self-sampling, and then transported the collected samples to county hospital for laboratory testing. | |
|  | *care*HPV (clinician-sampling) | Yes, supplies and equipment | Yes | Colposcopy on the same day | | Cervical sample was taken by clinician in county hospital, where all the clinical and laboratory process occurred. | |
|  | Full colposcopy, without biopsy | Yes, equipment | No | Directed biopsy and/or ECC (as required) | |  | |
|  | Biopsy / ECC | Yes, equipment | Yes | Treatment on next visit | | Biopsy was taken only after positive and satisfactory colposcopy examination; ECC only after negative and unsatisfactory (when the original squamocolumnar junction is not fully visible) colposcopy; and biopsy + ECC after positive and unsatisfactory colposcopy. | |
| **B. Type of treatment** | | **Indicated cervical disease** | **Number of bed days** | **Number of examinations /tests†** | **Type of anaesthesia (percentage)** | **Post-treatment follow-up (FU) management in the local hospitals** | |
| **At county level** | | | | | | | |
|  | LEEP | CIN2-3 | 1 | 17 | Local (100%) | | FU in one week after treatment using general gynecologic examination, then repeat in one month and 3 months if negative results, colposcopy FU in 6 months. |
|  | Cold-knife conization | CIN2-3 | 7 | 17 | Epidural (100%) | | FU in 3 months after treatment using general gynaecologic examination, and then Fu in 6 months using pap smear or general gynaecologic examination and ultrasonic examination. |
|  | Simple hysterectomy | CIN3-FIGOIa | 7 | 17 | Epidural (100%) | | FU in 3 months after treatment using general gynaecologic examination, and then FU in 6 months using Pap smear or general gynaecologic examination and ultrasonic examination. |
| **At prefecture level** | | | | | | | |
|  | Cold-knife conization | CIN | 7 | 12 | General (35%); Epidural (24%); Intrathecal (41%) | | FU in 3 months after treatment using cytology testing, and biopsy if necessary, then half-yearly FU over 2 years using Pap and colposcopy examination. |
|  | Simple hysterectomy | CIN3-FIGOIa | 9 | 17 | General (69%); Epidural (19%); Intrathecal (12%) | | FU in 3 months after treatment using general gynaecologic examination, followed by annual FU. |
|  | Radical hysterectomy | FIGOIb-FIGOIIa | 15 | 21 | General (100%) | | FU in one month after treatment using general gynaecologic examination, then FU in 3-6 months using Pap smear and ultrasonic examination (80%), followed by annual FU using pap smear and ultrasonic examination. |
|  | Simple radiotherapy | FIGOIIb+, part of FIGO I-IIa | 46 | 14 |  | |  |
|  | Neoadjuvant chemotherapy | FIGO IIB+ (locally advanced cervical cancer) | 3 | 6 |  | | FU in month 3, month 6 and month 12 after treatment using general gynaecologic examination, Pap smear and ultrasonic examination if needed; followed by half-yearly FU over 3 years, annual FU and back to normal population if negative result. |
|  | Adjuvant chemotherapy | Post-hysterectomy or post-radiotherapy | 9 | 16 |  | | FU in month 3, month 6 and month 12 after treatment using general gynaecologic examination, Pap smear and ultrasonic examination if needed; followed by half-yearly FU over 3 years, annual FU and back to normal population if negative result. |

† Including a range of examinations and tests of different costs, the number of histology examinations was equal to the number of sampled tissues (≥3).
